# A trial of consent procedures for future research with clinically derived biological samples

**DOI:** 10.1038/sj.bjc.6605339

**Published:** 2009-09-29

**Authors:** E Vermeulen, M K Schmidt, N K Aaronson, M Kuenen, M-J Baas-Vrancken Peeters, H van der Poel, S Horenblas, H Boot, V J Verwaal, A Cats, F E van Leeuwen

**Affiliations:** 1 The Netherlands Cancer Institute, Plesmanlaan 121, Amsterdam 1066 CX, The Netherlands

**Keywords:** residual tissue, scientific research, consent procedure, ownership

## Abstract

**Background:**

The aims of this study were to determine which consent procedure patients prefer for use of stored tissue for research purposes and what the effects of consent procedures on actual consenting behaviour are.

**Methods:**

We offered 264 cancer patients three different consent procedures: ‘one-time general consent’ (asked written informed consent), ‘opt-out plus’ (had the opportunity to opt out by a form), or the standard hospital procedure (control group). The two intervention groups received a specific leaflet about research with residual tissue and verbal information. The control group only received a general hospital leaflet including opt-out information, which is the procedure currently in use. Subsequently, all patients received a questionnaire to examine their preferences for consent procedures.

**Results:**

In all, 99% of patients consented to research with their residual tissue. In the ‘one-time consent’ group 85% sent back their consent form. Patients preferred ‘opt-out plus’ (43%) above ‘one-time consent’ (34%) or ‘opt-out’ (16%), whereas 8% indicated that they did not need to receive information about research with residual tissues or be given the opportunity to make a choice.

**Conclusions:**

The ‘opt-out plus’ procedure, which places fewer demands on administrative resources than ‘one-time consent’, can also address the information needs of patients.

Storage of human tissue after clinical procedures is important for two major reasons. First, the tissue can be used for future diagnosis and treatment of the patient and, second, it is a valuable resource for medical research (Ashburn *et al*, 2000; van Diest, 2002; Oosterhuis *et al*, 2003; Weir and Olick, 2004). Consent for use of stored tissue for research purposes can be obtained in a variety of ways, and there is on-going discussion about which procedure complies best with patients’ wishes while also being feasible in clinical practice (Maschke, 2005). Some argue that implied consent with the opportunity to opt-out is sufficient (van Diest, 2002; Furness, 2003; Coebergh *et al*, 2006; Bryant *et al*, 2008). Under the condition of ‘opt-out’, information about research with tissue is provided but patients are not actively asked to make a decision about research with their tissue. Others claim that explicit ‘one-time general’ (for all future research) consent should be asked (Hansson *et al*, 2006; Pentz *et al*, 2006; Wendler, 2006). An alternative for ‘one-time general consent’ is ‘one-time specific consent’ (providing consent for specific types of future research at one moment) that provides opportunities to have more control over future uses of tissue, but would stifle research (Greely, 1999; Lauri, 2002; Caulfield *et al*, 2003; Furness and Nicholson, 2004; Dickenson, 2007; Helft *et al*, 2007) or requires additional resources (Wheeler *et al*, 2007). Still others argue that informing patients about future research with tissue is impossible, even in basic terms, and that consent cannot be truly ‘informed’ (Hoeyer *et al*, 2004).

According to the Dutch Medical Treatment Act (Van Veen *et al*, 2006), anonymous samples obtained during medical treatment may be used in medical research if the patient has not objected to this ‘secondary use’. Active consent for use of such materials is not required and the law does not specify how patients should be informed about secondary use of tissue. The Medical Treatment Act does not regulate the use of coded samples. This is done by a Code of Conduct issued by the Dutch Federation of Medical Scientific Societies, conceived together with patient groups and the Royal Dutch Medical Association (Federation of Medical Scientific Societies, 2002). The need for a new law about the use of tissue for research is currently being discussed. Although some health attorneys argue that expressed or explicit consent should be obtained (Gevers, 2004; Olsthoorn-Heim and Gevers, 2004), others favour an opt-out procedure (Van Veen *et al*, 2006).

There are only very few published studies comparing the effects of informed consent procedures for the use of residual tissues for research purposes. In the international literature and in the Dutch context, the debate focuses on two procedures; ‘one-time consent’ or ‘opt-out’, that is, should patients be actively asked to consent or not? In earlier research of our group (Vermeulen *et al*, 2009a, 2009b), patients indicated that ‘opt-out’ would suffice if it was ascertained that patients were well informed. The respondents in these studies suggested a procedure that was labelled ‘opt-out plus’: patients receive verbal and written information, a special leaflet about tissue and research, and have the option to opt-out. In this study, ‘one-time consent’ and ‘opt-out plus’ were compared with the ‘opt-out’ procedure currently in use in the Netherlands to determine which consent procedure patients prefer for use of stored tissue for research purposes and what the effects of consent procedures on actual consenting behaviour are. The preference of patients for a specific consent procedure was evaluated through a follow-up survey and interviews.

## Materials and methods

The study was performed at the Netherlands Cancer Institute–Antoni van Leeuwenhoek Hospital (NKI–AVL) from August 2007 until January 2008. The NKI–AVL is a specialised cancer treatment hospital with a relatively small number of beds (180) and a large outpatient clinic.

**Figure 1:**
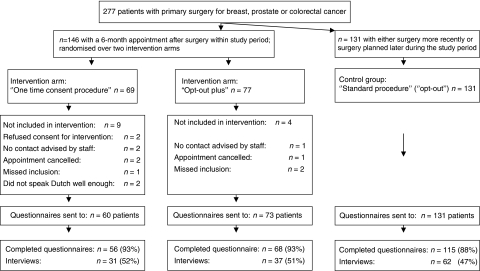
Study diagram: study groups, inclusion and response rates (questionnaires and interviews).

All patients who were younger than 75 years of age who had undergone primary surgery for breast, prostate or colorectal cancer (three of the major cancer types treated in the NKI–AVL) and were scheduled for an appointment within the time frame of the study, were selected from the hospital registry (*n*=277). We selected patients younger than 75 years because of the extra information load for the patients related to the research. Eligible patients who had a scheduled appointment at the outpatient clinic 6 months post-surgery, a standard follow-up appointment at the NKI–AVL for the selected tumour types, were randomly assigned to either the ‘one-time consent’ procedure or the ‘opt-out plus’ procedure (Figure 1) (*n*=146). The remaining patients, who had either surgery more recently or surgery planned later during the study period, were assigned to the control group (*n*=131). In this way patients were randomly assigned to the control group, based on the timing of surgery during the study period. We chose to offer the intervention post-surgically so as to avoid burdening patients during early diagnosis or early treatment phase with such additional information.

Patients who were assigned to one of the intervention arms were asked whether they consented to the intervention taking place during the outpatient clinic visit. The two consent procedures were offered in blocks, 10 patients of each of the three diagnostic groups were offered the ‘opt-out plus’ procedure, then 10 patients were offered the ‘one-time consent’ procedure.

In the two intervention groups, patients received brief verbal information in person from their nurse practitioner or physician, and a leaflet about the use of stored tissue, which was specifically written for this study. For the ‘one-time consent’ group, this verbal information was: ‘tissue that was excised during your surgery is stored in this hospital for future diagnosis or treatment. This tissue may also be used in medical research, but this requires your consent. Please read this leaflet at home and send back the consent form within 1 month in the stamped return envelope indicating whether you consent to the use of your tissue for future medical research.’ For the ‘opt-out plus’ group, the verbal information provided was; ‘tissue that was excised during your surgery is stored in this hospital for future diagnosis or treatment. This tissue may also be used in medical research. Please read this leaflet at home. You may want to object to research with your tissue and you may do so by sending back the form in the stamped return envelope.’ The special leaflets were four pages in length and contained paragraphs on storage and use of tissue in future research. Information about incidental findings and contact details in case of further questions was also included. The leaflets for the two intervention groups differed in wording ‘consent’ *vs* ‘opt-out’. The interventions were observed by one of the authors (EV). Patients received, together with the leaflet and the consent or opt-out form, a return envelop, which was addressed to the research team in the hospital. Patients who did not return the form were not sent a specific reminder but reminders were sent for the questionnaires.

The control group had received a general hospital leaflet according to the standard hospital procedures, which includes the general hospital leaflet of the NKI–AVL that informs patients, among others, about the possible use of stored tissues for research purposes. The text devoted to research with residual tissue in this leaflet is 170 words in length. It states that ‘residual tissue is sometimes used, as is the patients’ medical data, in anonymous and coded research. Patients are informed that they can opt-out if they wish by informing their physician, who will then make a note in the medical charts.’ This information policy is in line with Dutch law and comparable to that of other academic hospitals in the Netherlands.

A questionnaire was mailed to the patients in the intervention groups 4 weeks after the intervention took place. The patients in the standard procedure control group were sent a similar questionnaire 6 weeks post-surgery. The questionnaire was sent 6 weeks post-surgically because this would resemble the information time frame of the intervention patients while creating still somewhat more distance from the moment of diagnosis and surgical treatment. One month after the questionnaires had been mailed, non-respondents received a reminder. Patients who did not respond after the reminder were contacted by phone after 1 month.

The questionnaire covered a range of topics, including: reasons for (not) consenting, attitudes toward having been given the choice about tissue use, the perceived usefulness of the information provided in the leaflet, opinions about the best procedure for informing patients and for requesting consent from patients, and attitudes towards genetic and commercial research, tissue storage and ownership of tissue.

The question about the various consent procedures was introduced by text explaining that one-time general consent is a model in which the patient is informed and actively asked for written permission for all future research with archived tissue. Opt-out was described as the model in which the patient is not actively informed and not actively asked for consent; information about research with tissue is available in the general leaflet of the hospital and the patient can opt-out if (s)he so chooses. ‘Opt-out plus’ was described as the model in which patients are actively informed and offered the choice whether to opt-out of future research with their tissue. We also asked if patients would prefer repeated ‘fresh’ consent for specified research projects.

All patients were also asked to participate in a telephone interview. During these interviews a subset of questions from the written questionnaire was discussed in-depth, with particular emphasis placed on the questions about consent procedures, how patients perceived the storage and use of tissue, and types of research for which patients wished to give consent. If the respondent agreed, the interviews were audio recorded and subsequently transcribed. The study was approved by the hospital's institutional review board.

### Statistical analysis

Analyses were performed to determine the consent rates by consent procedure (based on the returned consent and opt-out forms), to compare the baseline characteristics of the three groups, and to compare the preference for and appreciation of the procedures (information obtained by questionnaire and interview) between the three groups (two intervention groups and the control group) and between subgroups formed on the basis of tumour type, gender, and educational level. Pearson's *χ*^2^-test was used to evaluate the significance of the differences. Significant predictors of preferences observed in logistic regression models at the univariate level were subsequently included in a multinomial logistic regression model. All statistical tests were two-sided, with a *P*-value of 0.05 or lower indicating statistical significance. The statistical software package SPSS 15.0 (SPSS Inc., Chicago, IL, USA) was used.

## Results

### Patient characteristics

Of the 146 eligible patients with primary surgery for breast, prostate or colorectal cancer, identified for block randomisation over the intervention groups, 133 (91%) were either given the ‘one-time consent’ procedure (*n*=60, 45%) or the ‘opt-out plus’ procedure (*n*=73, 55%) (Figure 1). Figure 1 shows that some patients could not be included in the study, for example, because they refused consent for the intervention or because the nurse practitioners advised not to approach him/her for the study because they considered the patient to have too much disease-related stress. Questionnaires were obtained from 56 patients (93%) in the ‘one-time consent’ group and 68 (93%) in the ‘opt-out plus’ intervention (Figure 1). From the 131 patients included in the control group, 115 (88%) questionnaires were obtained. In total, we obtained 239 of 277 (86%) questionnaires and 130 (47%) patients consented to an interview. Characteristics of patients in the three groups are shown in Table 1. Women were underrepresented in the opt-out plus group compared with the other groups (*P*=0.01). Distributions of age, sex and type of cancer were not significantly different between respondents and non-respondents (data not shown).

### Consent

Fifty-one consent forms (85%) were received from the 60 patients in the ‘one-time consent’ group. Most of these consent forms (*n*=45, 88%) were returned within 1 month after the intervention. None of the patients in the intervention groups withheld consent through the consent or ‘opt-out’ form. Though of the 73 patients randomised to the ‘opt-out plus’ intervention, two (3%) returned the ‘opt-out form’, during the interview it became clear that neither of these two patients was actually opposed to research with their tissue, but rather had misunderstood the directions regarding return of the form. Two respondents, one from the ‘one-time consent’ intervention and one from the ‘control group’, indicated in the follow-up questionnaire that they had actually wished to withhold consent for the use of tissue. Of all respondents, seven indicated in the questionnaire that they did not know whether they would want to ‘opt-out’.

The follow-up questionnaires and interviews showed that the primary reason for respondents to provide consent was a desire to contribute to improving treatment for future patients. Many respondents also stated that they expected that they or their relatives might personally benefit from future research that used their tissue.

### Appreciation of information

The majority of respondents (82 and 65% in the ‘one-time consent’ and ‘opt-out plus’ group, respectively) expressed appreciation at being informed about research with remaining tissue (Table 2). A minority of respondents felt that they were not well informed, but that they wished they had been informed (Table 2). Respondents in the ‘one-time consent’ group expressed more appreciation for the way they were informed by the hospital than respondents in the ‘opt-out plus’ group or the ‘control group’ (Table 2). Appreciation of information was not associated significantly with patients’ age, sex, educational level or diagnosis (data not shown). Of the respondents in the control group, 73% had not seen or did not remember having read the information about tissue and research in the general hospital leaflet. Providing information to patients in the two intervention groups took little time (approximately 1 min). Patients rarely asked questions about tissue and research (in later contacts with staff members).

### Preferences for consent procedures

Based on all groups combined, 43% of respondents preferred ‘opt-out plus’, 16% ‘opt-out’, 34% ‘one-time consent’, and 8% ‘no information at all’ (Table 3). The majority of respondents preferred information to be given before or during hospitalisation (Table 3). Preferences for the various consent procedures did not differ as a function of the intervention group.

Based on the univariate analyses, preference for a regimen varied significantly as a function of age, sex, educational level and ownership feelings (that is, the respondent felt that the stored residual tissue was still a part of him/herself after removal). Respondents who were younger, female, more highly educated and considered themselves owner of their tissue or the DNA in their tissue, were significantly more likely to prefer the ‘one-time consent’ and ‘opt-out plus’ procedures to ‘opt-out’. In all subgroups, the ‘opt-out plus’ procedure was preferred more often than the other consent procedures (Table 4) .

In the multinomial logistic regression analysis, education and ownership attitudes remained significant predictors of preference for ‘opt-out plus’ to ‘opt out’ (Table 5). These two factors did not explain preference for ‘one-time consent’ compared with ‘opt-out plus’.

### Expectations of patients regarding the use of residual tissues in research

Most respondents (98%) trusted (hospital) regulations and the protection of privacy (82%), but many (49%) were opposed to the use of their residual tissues in ‘commercial research’. Those who were opposed to the use of their tissue in ‘commercial research’ tended to be more highly educated than those who had no such objections (*P*=0.008). During the interviews, many respondents stressed the need for research results to become available for the benefit of future patients; they considered use of tissues by commercial parties as not contributing to the common good.

On the basis of a feeling of reciprocity, the majority of respondents (72%) expected to be informed about research findings based on the use of their tissue. This typically reflected an interest in science in general, but for some it was a desire to be able to check the type of research for which their tissue had been used. Most respondents (91%) also wanted to be informed about any incidental findings that might be relevant to their treatment.

## Discussion

To our knowledge, this study is the first to compare different consent procedures for research with residual tissue. We examined the effects of three different procedures on consent rates and patients’ attitudes. Nearly all patients (99%) consented to research with residual tissue. We did not find higher rates of withdrawal of consent as a result of specific consent regimens. Of the patients in the ‘one-time consent’ group, most returned the consent form as requested. Patients were significantly more satisfied with the way information was provided about research with residual tissues in the ‘one-time consent’ and ‘opt-out plus’ groups, compared with the control group. In the follow-up questionnaire, patients preferred ‘opt-out plus’ above ‘one-time consent’ or ‘opt-out’ without actively being informed. Younger, more highly educated patients and patients who believed that they ‘owned’ their tissue were significantly more likely to prefer ‘one-time consent’ above ‘opt-out’, but not above ‘opt-out plus’.

Studies in other settings have reported similarly high consent rates for the use of residual tissue in medical research (Hamajima *et al*, 1998; Malone *et al*, 2002; Stegmayr and Asplund, 2002; Jack and Womack, 2003; Furness and Nicholson, 2004; Chen *et al*, 2005; Wheeler *et al*, 2007; Bryant *et al*, 2008). Our results show that patients prefer to be informed about research with tissue, refuting authors who argue that patients do not need to be informed and do not need to be provided with the opportunity to opt-out or withhold consent for future research with residual tissue (Keulartz *et al*, 2004; Swierstra, 2004). Respondents felt respected and valued by being informed, but most felt that actually giving consent was of secondary importance (66% did not prefer ‘one-time consent’), as was also observed by Hamilton *et al* (2007) and previous studies of our own group (Vermeulen *et al*, 2009a, 2009b). The consent procedure that was offered to patients in the ‘one-time consent’ and ‘opt-out plus’ groups involved the reading of several pages of information in the leaflet and we asked the patient to read this leaflet at home.

We considered it undesirable to ask patients to read the information and make a decision about consent or opt-out concerning research with tissue immediately during the consultation with the staff member. According to national and international ethical guidelines, patients should have several days to consider their decision to sign a consent form or not. It is possible that some patients would prefer to make a decision during the consultation (though none of the patients specifically indicated this). In such a scenario patient preferences could differ from our findings and such an ‘in-person’ procedure would eliminate part of the additional work required for the one-time consent compared with the opt-out-plus procedure. Some patients indicated in the interview that they deemed the additional paper administration of the informed consent procedure unnecessary. However, none declared that this was the main reason for preferring the opt-out plus above the informed consent procedure.

Several authors have argued that implied consent with ‘opt-out’ suffices (van Diest, 2002; Coebergh *et al*, 2006; Johnsson *et al*, 2008a); they fear that a ‘one-time consent’ regimen would delay or inhibit research or that the informed consent process would itself cause selection bias (Junghans *et al*, 2005; Halamka, 2006; Watson, 2006). The report of the Dutch Royal Academy of Sciences (Royal Dutch Academy of Sciences. Foresight Committee on Multifactorial Diseases in the Genomics Age, 2006) also advised ‘opt-out’, largely based upon concerns that obtaining ‘one-time general consent’ would involve a great deal of effort from health-care workers, and that nonetheless a substantial non-response would result, which would create a bias in the tissue collections available for research (Junghans *et al*, 2005; Helft *et al*, 2007). We observed no negative effect of the interventions in the sense that more patients withheld consent. However, only 85% of patients asked for ‘one-time consent’ returned the consent form (without a reminder). This implies that the model of ‘one-time consent’ would cost considerable effort from the hospital to obtain higher response rates. We found that a brief verbal explanation by the nurse practitioners or physician of the ‘opt-out plus’ procedure was feasible and appreciated by patients. This was also suggested by others (Axler *et al*, 2008; Johnsson *et al*, 2008b; Hewitt *et al*, 2009). Others found indications that less privileged groups might be more reluctant to research with tissue (Helft *et al*, 2007) and may want to be asked for consent in a stricter procedure. We think, however, that the opt-out plus procedure is also strict seen from the patients’ perspective; the patient is as fully informed as in the one-time consent procedure and is facilitated to make a decision. We think therefore that opt-out plus could also be a suitable procedure in populations or countries less trusting. However, when obtaining samples explicitly for research purposes only, explicit consent of patients is required (also according to Dutch law).

When interpreting the results of our study, its strengths and limitations should be considered. The study groups were relatively small and only few patients withheld the consent, which did not allow subgroup analysis of decliners. Our study population was restricted to cancer patients diagnosed before the age of 76 years who were treated in a specialised cancer hospital. Thus, it is possible that the observed preferences for the three consent regimens might be different in (non-) cancer patient populations treated in general, community hospitals. Older and less educated patients more often preferred regimens without active provision of information. As the cancer patients treated in our hospital tend to be somewhat younger and more highly educated than those treated in community hospitals, one might expect that, in other treatment settings, even more patients would prefer the ‘opt-out plus’ procedure.

We have not measured how strong patients’ opinions for a specific consent procedure were. As most patients stressed the importance of informing patients, we think that this is the most important element of the procedure. We suppose that patients who feel that they want to be asked for explicit consent can see this procedure as a means of asking consent, while others who do not care about their residual tissue, can easily put the information aside.

Patients in our study considered their tissue to be special, as it allows them to contribute to medical progress. Few empirical data exist describing the attitudes of cancer patients towards residual tissue storage and use in research (Kaphingst *et al*, 2006). Earlier studies found that surgically resected tissue had no special emotional value for most respondents (Start *et al*, 1996; Medical Research Council, 2000). However, a sizeable minority (38%) of our respondents considered themselves to be owners of residual tissue. In other research only 10% (Start *et al*, 1996) or 23% (Bryant *et al*, 2008) of patients stated that they believed they retained ownership over tissue removed at surgery. However, our respondents did not interpret ‘ownership’ legally. They considered the tissue to be theirs, but they did not feel they should derive rights from research with their tissue. Many patients expected to be informed about research findings based on the use of their tissue in research. How this should or could be done is an important issue because it is associated with a positive attitude of patients towards research (Kaufman *et al*, 2008). The respondents felt it was important that residual tissues be used for the common good; for many this meant they did not want to donate tissues for all future uses. In particular, ‘commercial use’ raised questions, and many respondents preferred that their tissue should not be used commercially. Some studies report wariness about ‘commercial use’ while other studies do not (Jack and Womack, 2003; Treweek *et al*, 2009). Commercial use may deserve more attention in regulatory aspects and information to patients, because a large part of research is taking place in an environment in which ‘commercial parties’ are involved. A good understanding of patients may be crucial for patient cooperation.

In conclusion, our results suggest that cancer patients prefer an ‘opt-out plus’ procedure for consenting to medical research with residual tissue. This includes both a brief, verbal explanation and written materials that can be read at home. This approach represents a feasible, intermediate procedure between ‘opt-out’, which leaves many patients uninformed, and ‘one-time general consent’, which burdens hospital staff and may create bias in tissue collections. We think that this procedure may unify two moral principles stressed by our respondents: that patients are informed and that medical research can progress without unnecessary hindrance.

## Figures and Tables

**Table 1:** Characteristics of respondents of the questionnaires in the three study groups

	**One-time consent study group (*n*=56)**	**Opt-out plus study group (*n*=68)**	**Control study group (*n*=115)**	**All respondents (*n*=239)**	**Non response (*n*=38)**
Age (at time of study) *P-value=0.14					
<50 years	11 (20%)	9 (13%)	30 (26%)	50 (21%)	12 (32%)
50–64 years	29 (52%)	40 (59%)	65 (57%)	134 (56%)	16 (42%)
65–75 years	16 (29%)	19 (28%)	20 (17%)	55 (23%)	10 (26%)
					
Sex *P-value=0.01					
Female	28 (50%)	29 (43%)	74 (64%)	131 (55%)	18 (47%)
Male	28 (50%)	39 (57%)	41 (36%)	108 (45%)	20 (53%)
					
Cancer diagnosis *P-value=0.06					
Breast cancer	21 (38%)	20 (29%)	57 (50%)	98 (41%)	15 (40%)
Prostate cancer	18 (32%)	27 (40%)	26 (23%)	71 (30%)	10 (26%)
Colorectal cancer (female)	7 (12%)	9 (13%)	17 (15%)	33 (14%)	4 (10%)
Colorectal cancer (male)	10 (18%)	12 (18%)	15 (13%)	37 (15%)	9 (24%)
					
Educational level (a) *P-value=0.28					
Low	13 (23%)	7 (10%)	20 (17%)	40 (17%)	NA
Intermediate	21 (38%)	36 (53%)	56 (49%)	113 (47%)	NA
High	22 (39%)	25 (37%)	39 (34%)	86 (36%)	NA

(a) Low level education: primary school, lower vocational training or lower general training; intermediate level: intermediate vocational or intermediate/higher general; high level: higher vocational or university training.

^*^Differences tested between the three study groups.

**Table 2:** Study groups and appreciation of information

	**One-time consent study group**	**Opt-out plus study group**	**Control study group**
Appreciation of information *P-value=0.00			
I was well informed (*n*=114, 51%)	82%	65%	27%
Information was neither good nor bad (*n*=68, 31%)	15%	29%	40%
I was poorly informed (*n*=41, 18%)	4%	6%	33%

^*^Differences tested between the three study groups.

**Table 3:** Preferences regarding consent procedure for research use of residual tissue

	**All respondents (*n*=239)**
*Type of consent procedure*	
‘One-time consent’ is the appropriate consent procedure	81 (34%)
‘Opt-out plus’ is the appropriate consent procedure	103 (43%)
‘Opt-out’ is the appropriate consent procedure	37 (16%)
No information needs to be provided	18 (8%)
	
*When should information be provided?*	
I think it should be given before or at intake	93 (39%)
I think it should be provided during the hospital stay	50 (21%)
I think it should be provided after the hospital stay	69 (29%)
I think it should be offered otherwise	28 (11%)
	
*How should written information be provided?*	
In a general hospital leaflet	100 (42%)
In a specific leaflet about residual tissue and research	119 (50%)
I think no information should be provided	12 (5%)
Information should be provided otherwise	6 (3%)

**Table 4:** Predictors of preference for different consent procedure

	**Preference reported in the questionnaire**				
	**Prefer one-time consent**	**Prefer opt-out plus**	**Prefer opt-out**	**Information not needed**	*P*-value**
*Study group*					0.53
One-time consent (*n*=56)	38%^*^	45%^*^	9%^*^	9%^*^	
Opt-out plus (*n*=68)	35%	37%	18%	10%	
Control group (*n*=115)	31%	46%	17%	5%	
					
*Age (at time of study)*					0.008
<50 years (*n*=50)	40%	40%	18%	2%	
50–64 year (*n*=134)	37%	45%	13%	5%	
65–75 years (*n*=55)	20%	42%	20%	18%	
					
*Sex*					0.46
Female (*n*=131)	38%	41%	14%	7%	
Male (*n*=108)	29%	45%	18%	8%	
					
*Cancer diagnosis*					0.86
Breast cancer (*n*=98)	39%	42%	13%	6%	
Prostate cancer (*n*=71)	32%	44%	16%	9%	
Colorectal cancer (*n*=70)	29%	44%	19%	9%	
					
*Educational level (a)*					0.01
Low (*n*=40)	20%	35%	28%	18%	
Intermediate (*n*=113)	35%	43%	15%	7%	
High (*n*=86)	38%	48%	11%	4%	
					
*Do you consider yourself the owner of stored tissue?*					0.039
No (*n*=144, 62%)	29%	42%	20%	9%	
Yes (*n*=87, 38% )	41%	45%	8%	6%	
					
*Do you consider yourself the owner of DNA in stored tissue?*					0.006
No (*n*=111, 47%)	32%	37%	19%	12%	
Yes (*n*=98, 43%)	36%	53%	7%	4%	

(a) Low level education: primary school, lower vocational training or lower general training; intermediate level: intermediate vocational or intermediate/higher general; high level: higher vocational or university training.

^*^Row percentages count to 100. ^**^Differences tested between the four preferences reported in the questionnaire.

**Table 5:** Predictors of preference for one-time consent or opt-out using opt-out plus as a reference category in a multivariate model using multinomial logistic regression

	**HR (95% CI)**	***P*-value**
*One-time general consent* a		
Age		
<50	1.77 (0.65–4.86)	NS
50–65	1.68 (0.71–3.94)	NS
>60	Ref	
		
*Educational level*		
Low	0.73 (0.24–2.22)	NS
Intermediate	0.97 (0.50–1.88)	NS
High	Ref	
		
*Do you consider yourself owner of residual tissue?*		
No	0.64 (0.34–1.22)	NS
Yes	Ref	
		
*Opt-out* a		
Age		
<50	1.73 (0.51–5.89)	NS
50–65	0.65 (0.23–1.86)	NS
>65	Ref	
		
*Educational level*		
Low	4.70 (1.44–15.39)	0.01
Intermediate	1.54 (0.56–4.26)	NS
High	Ref	
		
*Do you consider yourself the owner of residual tissue?*		
No	3.60 (1.30–9.97)	0.01
Yes	Ref	

a Opt-out plus=reference category.
